# Activation of Type 1 Cannabinoid Receptor (CB1R) Promotes Neurogenesis in Murine Subventricular Zone Cell Cultures

**DOI:** 10.1371/journal.pone.0063529

**Published:** 2013-05-21

**Authors:** Sara Xapelli, Fabienne Agasse, Laura Sardà-Arroyo, Liliana Bernardino, Tiago Santos, Filipa F. Ribeiro, Jorge Valero, José Bragança, Clarissa Schitine, Ricardo A. de Melo Reis, Ana M. Sebastião, João O. Malva

**Affiliations:** 1 Center for Neuroscience and Cell Biology of Coimbra, University of Coimbra, Coimbra, Portugal; 2 Institute of Pharmacology and Neurosciences, Faculty of Medicine, University of Lisbon, Lisboa, Portugal; 3 Unit of Neurosciences, Instituto de Medicina Molecular, University of Lisbon, Lisboa, Portugal; 4 Health Sciences Research Center, University of Beira Interior, Covilhã, Portugal; 5 Institute for Biotechnology and Bioengineering, Centre for Molecular and Structural Biomedicine, University of Algarve, Faro, Portugal; 6 Neurochemistry Laboratory, Biophysics Institute Carlos Chagas Filho, Federal University of Rio de Janeiro, Rio de Janeiro, Brazil; 7 Center for Research on Environment, Genetics and Oncobiology (CIMAGO), Faculty of Medicine (polo 3), University of Coimbra, Coimbra, Portugal; University of Nebraska Medical Center, United States of America

## Abstract

The endocannabinoid system has been implicated in the modulation of adult neurogenesis. Here, we describe the effect of type 1 cannabinoid receptor (CB1R) activation on self-renewal, proliferation and neuronal differentiation in mouse neonatal subventricular zone (SVZ) stem/progenitor cell cultures. Expression of CB1R was detected in SVZ-derived immature cells (Nestin-positive), neurons and astrocytes. Stimulation of the CB1R by (R)-(+)-Methanandamide (R-m-AEA) increased self-renewal of SVZ cells, as assessed by counting the number of secondary neurospheres and the number of Sox2+/+ cell pairs, an effect blocked by Notch pathway inhibition. Moreover, R-m-AEA treatment for 48 h, increased proliferation as assessed by BrdU incorporation assay, an effect mediated by activation of MAPK-ERK and AKT pathways. Surprisingly, stimulation of CB1R by R-m-AEA also promoted neuronal differentiation (without affecting glial differentiation), at 7 days, as shown by counting the number of NeuN-positive neurons in the cultures. Moreover, by monitoring intracellular calcium concentrations ([Ca^2+^]_i_) in single cells following KCl and histamine stimuli, a method that allows the functional evaluation of neuronal differentiation, we observed an increase in neuronal-like cells. This proneurogenic effect was blocked when SVZ cells were co-incubated with R-m-AEA and the CB1R antagonist AM 251, for 7 days, thus indicating that this effect involves CB1R activation. In accordance with an effect on neuronal differentiation and maturation, R-m-AEA also increased neurite growth, as evaluated by quantifying and measuring the number of MAP2-positive processes. Taken together, these results demonstrate that CB1R activation induces proliferation, self-renewal and neuronal differentiation from mouse neonatal SVZ cell cultures.

## Introduction

In the adult brain, the SVZ is endowed with neural stem cells that give rise to highly proliferating progenitor cells, able to differentiate into neurons and glial cells [1], [2]. *In vivo*, the progenitors undergo cell death [3] or originate neuroblasts that migrate tangentially along the rostral migratory stream (RMS) towards the olfactory bulb [4] where they become functional and integrate into pre-established brain circuits [5]. In addition, in a different perspective, brain injuries, such as seizures, head traumas and stroke, result in stimulation of neurogenesis, and this has been proposed as an endogenous attempt to repair and reduce brain damage [6]. In fact, newly generated cells migrate out of the SVZ towards the damaged areas upon several brain injuries and neurodegenerative diseases [7], [8]. Therefore, these proliferative and multipotent cells may represent a potential source of neurons and glia for brain repair, through the recruitment from the endogenous niches or through transplantation strategies [9], [10].

Although the knowledge regarding neurogenesis is increasing, the factors available at stem cell niches that may regulate cell proliferation, differentiation, survival, maturation and integration remain poorly understood. Deciphering the molecular mechanisms controlling these events will contribute to the development of new strategies to treat brain diseases.

There is an emerging consensus that endocannabinoid signaling plays a major role in adult neurogenesis. Cannabinoids act on at least two types of receptors, the type 1 and type 2 cannabinoid receptors (CB1R and CB2R), which are, respectively, predominantly distributed in the central nervous system (CNS) and immune system, although some studies have described the presence of low levels of CB2R in the brain [11], [12]. In the brain, CB1R are targeted by endogenous cannabinoids (or endocannabinoids) such as anandamide (AEA) and 2-arachidonylglycerol (2-AG), which are molecules generated by the cleavage of plasma membrane lipid precursors, a reaction tightly controlled by neuronal activity [13]. Once generated, endocannabinoids act retrogradely through presynaptic CB1R, blunting membrane depolarization and inhibiting neurotransmitter release [13]. Collectively, CB1R agonists render neurons less excitable and thus promote neuroprotection [14]. The endocannabinoid system has been proposed to play important roles in many pathophysiological processes such as Parkinson’s disease, Alzheimer’s disease, depression, inflammation, neuropathic pain and obesity [15], [16].

Several reports have demonstrated the modulation of neural stem cell proliferation in culture and/or in adult mice *via* CB1R and CB2R activation [17]–[23]. Additionally, *in vivo* studies showed that excitotoxicity-induced hippocampal neural progenitors proliferation and neurogenesis are abolished in CB1R-knockout (KO) mice and in wild-type (WT) mice administered with a selective CB1R antagonist [24]. Moreover, cannabinoid receptor activation was found to promote migration of SVZ-derived neuroblasts [25].

Although recent data have highlighted the importance of endocannabinoids in neurogenesis, available studies in the field mostly addressed proliferation and did not analyse their influence on stem cell properties and neuronal differentiation. Therefore, we have dissected the effects of the agonist (*R*)-(+)-Methanandamide (R-m-AEA) on stem cell dynamic, proliferation, cell death, and progenitor’s neuronal differentiation in mouse SVZ cultures. Our data clearly show that CB1R activation has a proneurogenic effect on SVZ cells, suggesting that this pathway may be modulated in order to activate neurogenesis in SVZ cells.

## Materials and Methods

### Ethics Statement

All experiments were performed in accordance with the European Community (86/609/EEC; 2010/63/EU) guidelines for the care and use of laboratory animals. The work was performed with biological material obtained from mouse pups and subsequently maintained *in vitro*. The study was approved by the internal institutional ethic committee of the animal house (Biotério FMUC; License n°520.000.000.2006, from the Portuguese animal welfare authorities) after approval of the research project POCTI/SAU-NEU 68465/2006. Sara Xapelli is a Competent Authorised Person (Scientist- FELASA-CAT.C) for handling and conducting laboratory animals in scientific research. The pups were handled according to standard and humanitarian procedures to reduce animal suffering. The animals were sacrificed by decapitation and the brains immediately removed. Both WT C57BL6 and transgenic mice expressing the green fluorescent protein (C57BL/6-Tg(CAG-EGFP)1Osb/J, Jackson Laboratories, Maine, USA) were used.

### SVZ Cell Cultures

SVZ neurospheres were prepared from 1- to 3-day-old C57BL/6 mice in serum-free medium (SFM) supplemented with 10 ng/ml epidermal growth factor and 5 ng/ml fibroblast growth factor-2 (EGF and FGF-2; Invitrogen, Carlsbad, CA, USA), as described previously [26] (see Supporting Information). In fact, these conditions are optimal for the selection of stem/progenitor cells from SVZ tissue [27]–[29]. Using this protocol, obtained neurospheres are composed of undifferentiated cells expressing Sox2 and Nestin (Fig. S1). Six days after plating, the resulting SVZ neurospheres were seeded onto glass coverslips coated with 0.1 mg/ml poly-D-lysine in SFM medium devoid of growth factors. Two days after plating, the medium was renewed with or without (control) a range of concentrations for CB1R ligands.

### Pharmacological Treatments

To investigate the effect of R-m-AEA [(*R*)-*N*-(2-Hydroxy-1-methylethyl)-5*Z*,8*Z*,11*Z*,14*Z*-eicosatetraenamide] (CB1R agonist; Tocris, Ellisville, MO, USA) on cell proliferation, neurospheres plated as aforementioned were allowed to develop for 48 h in the absence (control) or in the presence of R-m-AEA (100 nM, 300 nM or 1 µM) and 10 µM of 5-bromo-2-deoxyuridine (BrdU) (Sigma-Aldrich, St Louis, MO, USA) was added for the last 4 h of the culture session. To investigate the influence of R-m-AEA on differentiation, neurospheres were allowed to develop for 7 days in the absence (control) or in the presence of R-m-AEA (100 nM, 300 nM or 1 µM) and/or CB1R antagonist AM 251 [*N*-(Piperidin-1-yl)-5-(4-iodophenyl)-1-(2,4-dichlorophenyl)-4-methyl-1*H*-pyrazole-3-carboxamide] (1 µM, Tocris). Neuronal differentiation of proliferating progenitors was assessed in SVZ cells dissociated from neurospheres and plated at a density of 50000 cells/cm^2^ onto poly-D-lysine coated glass coverslips in SFM for 48 h. The cultures were incubated in the absence (control) or in the presence of 1 µM R-mAEA together with 10 µM BrdU for 24 h. Then the cells were washed and incubated with only R-m-AEA for more 6 days, fixed and stained for a marker of mature neurons (neuronal nuclear protein, NeuN) and BrdU.

To study the influence of R-m-AEA on neuritogenesis, SVZ neurospheres obtained from WT and GFP transgenic newborn mice were dissociated (Neurocult dissociation kit, Stemcell Technologies Inc., Grenoble, France) and plated onto poly-D-lysine coated glass coverslips (95% WT and 5% GFP), in SFM devoid of growth factors at a density of 50000 cells/cm^2^. With this strategy we could differentiate high-density SVZ cells endowed with isolated GFP-positive cells. The cells were allowed to develop for 7 days in the absence (control) or in the presence of 1 µM R-m-AEA, and were then fixed and stained for microtubule-associated protein 2 (MAP2).

To access whether (*R*)-(+)-Methanandamide promotes trimethylation of histone H3 lysine 36 (H3K36m3) on the promoter region of Neurogenin1 (*Ngn1*), cells were incubated with 1 µM (*R*)-(+)-Methanandamide for 6 h and 24 h, and then processed for quantitative chromatin immunoprecipitation (qChIP) (see Methods S1). To confirm qChIP analysis, the levels of *Ngn1* mRNA were determined by quantitative real time polymerase chain reaction (qRT-PCR) in SVZ cells treated or not (control) with 1 µM R-m-AEA for 3 days (see Methods S1).

### Self-renewal and Multipotency Assay

Self-renewal assays were performed on SVZ cells seeded at clonal density, at 2500 cells per well in 24-well cell culture plates in SFM containing 5 ng/ml EGF and 2.5 ng/ml FGF-2 (low EGF/FGF-2) and supplemented or not (control) with 1 µM R-m-AEA and/or 10 µM DAPT (a γ-secretase inhibitor and therefore an inhibitor of Notch pathway). After 6 days, the number of primary neurospheres was determined. Then, neurospheres were collected, dissociated as single cells (Neurocult dissociation kit) and seeded in low EGF/FGF-2 medium as aforementioned. After 6 days, the number of secondary neurospheres was counted. Then the neurospheres were adhered to SuperFrost Plus glass slides (Thermo Scientific, Menzel GmbH & Co KG, Braunscheweig, Germany) by cytocentrifugation (360×g, 5 min; Cellspin I, Tharmac GmbH, Waldsoms, Germany) and the neurospheres were immunolabeled for Oligodendrocyte transcription factor 2 (Olig2), Glial fibrillary acidic protein (GFAP) and doublecortin (DCX).

### Cell-fate Studies: Sox2 Cell Pair Assay

Dissociated SVZ cell suspension obtained during the cell culture procedure was plated on poly-D-lysine coated glass coverslips at a density of 6400 cells/cm^2^. After seeding, SVZ cells were grown in low EGF/FGF-2 containing medium supplemented or not (control) with 1 µM R-m-AEA and/or 10 µM DAPT for 24 h. Thereafter, cells were fixed in methanol for 15 min at −20°C and then processed for immunocytochemistry against Sox2.

### Immunocytochemistry

Cells were fixed for 30 minutes in 4% paraformaldehyde in phosphate-buffered saline (PBS) or methanol, permeabilized and blocked for non-specific binding sites for 1 h with 0.25% Triton X-100 (Sigma-Aldrich) and 3% bovine serum albumin (BSA, Sigma-Aldrich) dissolved in PBS. Cells were then subsequently incubated overnight at 4°C with primary antibodies as listed in Table 1 and for 1 h at RT with the appropriate secondary antibodies as follows: donkey anti-mouse Alexa Fluor 594 antibody, anti-rabbit Alexa Fluor 488 or anti-goat Alexa Fluor 488 (all 1∶200 and all from Invitrogen). Nuclei were visualized after Hoechst 33342 incubation (6 µg/ml in PBS, Invitrogen). Finally, the preparations were mounted using Dakocytomation fluorescent medium (Dakocytomation, Carpinteria, CA, USA). Fluorescence images were recorded using an Axioskop microscope (Carl Zeiss Inc., Göttingen, Germany) and confocal images were recorded using a Zeiss LSM 510 META confocal microscope (Carl Zeiss Inc., Göttingen, Germany).

**Table 1:** Primary antibodies used for immunocytochemistry.

Antigen	Company	Catalog number	Host	Clonality	Dilution
**CB1R**	Proteimax (Cotia, Brazil)	PROTX07	rabbit	polyclonal	1∶200
**Glial fibrillary acidic protein (GFAP)**	Cell Signaling Technology (Danvers, MA, USA)	3670	Mouse	Monoclonal	1∶500
**Microtubule-associated** **protein 2 (MAP2)**	Sigma-Aldrich (St Louis, MO, USA)	M4403	Mouse	monoclonal	1∶200
**Doublecortin (DCX)**	Santa Cruz Antibodies, Santa Cruz, CA, USA)	sc-8066	Goat	polyclonal	1∶200
**Nestin**	Abcam (Cambridge, UK)	ab6142	Mouse	monoclonal	1∶200
**BrdU linked to IgG-labeled Alexa fluor 594**	Invitrogen (Invitrogen, Carlsbad, CA, USA)	A21304	Mouse	monoclonal	1∶100
**BrdU**	AbD serotec (Oxford, UK)	OBT0030	Rat	monoclonal	1∶50
**βIII tubulin**	Cell Signaling Technology	4466	Mouse	monoclonal	1∶500
**Vesicular GABA Transporter (VGAT)**	Synaptic Systems (Goettingen, Germany)	131011	Mouse	monoclonal	1∶200
**Neuronal Nuclei (NeuN)**	Millipore (Billerica, MA, USA)	MAB377	Mouse	monoclonal	1∶100
**anti-Tyrosine Hydroxylase** **(TH)**	Abcam (Cambridge, UK)	AB112	Rabbit	polyclonal	1∶100
**Olig2**	Millipore	AB9610	Rabbit	polyclonal	1∶200
**Sox2**	Santa Cruz Antibodies	sc-17320	Goat	polyclonal	1∶600
**Polysialylated neuronal** **cell adhesion molecule** **(PSA-NCAM)**	Millipore	MAB5324	Mouse	monoclonal	1∶200

### Cell Proliferation Studies

To investigate the effect of (*R*)-(+)-Methanandamide (R-m-AEA) on cell proliferation, SVZ cells were exposed to 10 µM 5-bromo-2′-deoxyuridine (BrdU) (Sigma-Aldrich), a synthetic thymidine analogue able to substitute thymidine in the DNA double chain synthesis occurring in dividing cells, for the last 4 h of each R-m-AEA (100 nM, 300 nM or 1 µM) treatment (48 h), as described previously [30]. Then, SVZ cells were fixed in 4% PFA for 30 min and rinsed with 0.15 M PBS at RT. Thereafter BrdU was unmasked by permeabilizing cells in PBS 1% Triton X-100 at RT for 30 min and DNA was denaturated in 1 M HCl for 40 min at 37°C. Following incubation in PBS with 0.5% Triton X-100 and 3% BSA to block nonspecific binding sites, cells were incubated overnight with the anti-BrdU antibody (Table 1). After an additional rinse in PBS, SVZ nuclei counterstaining and mounting were performed as described previously.

Proliferation was also assessed using the Elisa BrdU colorimetric assay (Roche, Basel, Switzerland). For that purpose, 40000 SVZ cells obtained from dissociated primary neurospheres (Neurocult dissociation kit) were plated per well of 96 well culture plates (4 wells per condition) and treated in the absence (control) or presence of 1 µM R-m-AEA in SFM devoid of growth factors. BrdU (10 µM) was added 4 h before the end of the 48 h lasting culture session. Amount of BrdU incorporation was evaluated by densitometry (at 450 nm) following the use of a peroxidase conjugated anti-BrdU antibody and reaction with a peroxidase substrate according to manufacturer’s instructions. As a positive control, 10 ng/ml EGF together with 5 ng/ml FGF-2 was used.

### Western Blotting Analysis

Western blotting analysis of CB1R, GFAP, Olig2 and phospho-extracellular-signal-regulated kinase 1/2 (P-ERK1/2) was performed from 6 day-old neurospheres that were plated into 6-well plates previously coated with 0.1 mg/ml poly-D-lysine, and that were allowed to adhere for 48 h in SFM before treatment or not (control) with R-m-AEA (1 µM) for 15 min and 30 min (for P-ERK) or 7 days (for CB1R, GFAP and Olig2). The cells were then washed with 0.15 M PBS and harvested by scraping in lysis buffer [0.15 M NaCl, 0.05 M Tris-base, 5 mM EGTA, 1% Triton X-100, 0.5% Sodium deoxycholate (DOC), 0.1% SDS, 10 mM dithiothreitol (DTT), containing a protease inhibitor cocktail tablet (Roche), pH 7.4 at 4°C]. The supernatant was collected after centrifugation at 14 000 rpm for 10 min, at 4°C. Protein concentration was measured by the BCA method and samples were treated with SDS-PAGE sample buffer [6× concentrated: 350 mM Tris, 10% (w/v) SDS, 30% (v/v) glycerol, 0.6 M DTT, 0.06% (w/v) bromophenol blue], boiled 5 min at 95°C, and stored at −20°C until use for Western blotting analysis. Then, proteins were separated by SDS-PAGE on 10% acrylamide/bisacrylamide gels and transferred onto PVDF (polyvinylidine difluoride) membranes with 0.45 µm pore size in the following conditions: 300 mA, 90 min at 4°C in a solution containing 10 mM CAPS and 10% methanol, pH 11. Membranes were blocked and incubated with the antibodies against CB1R (Proteimax, Cotia, Brazil), GFAP (Cell Signaling Technology, Danvers, MA, USA), Olig2 (Millipore, Billerica, MA, USA) or P-ERK1/2 (Cell Signaling Technology). After washing, membranes were incubated for 1 h at RT, with the respective alkaline phosphatase-linked secondary antibodies. For endogenous control of immunolabeling, PVDF membranes were reprobed with the antibodies against α-Tubulin (Millipore) for CB1R, β-actin (Abcam, Cambridge, UK) for GFAP and Olig2 and ERK1/2 (Cell Signaling Technology) for P-ERK1/2. Then the membranes were incubated with the alkaline phosphatase-linked secondary antibodies. Protein immunoreactive bands were visualized in a Versa-Doc Imaging System (model 3000, BioRad Laboratories, CA), following incubation of the membrane with ECF reagent (GE Healthcare, Buckinghamshire, UK) for 5 min. Densitometric analyses were performed by using the ImageQuant software (GE Healthcare Life Sciences).

### Single Cell Calcium Imaging (SCCI)

To determine the functional differentiation pattern of SVZ cells, the variations of [Ca^2+^]_i_ in single cells following stimulation with 50 mM KCl (Merck; Darmstadt, Germany) and 100 µM histamine (Sigma-Aldrich) were analyzed [31]. Histamine/KCl ratios of peak values for Fura-2 were calculated to determine the extent of neuronal differentiation in SVZ cultures (see Methods S1).

### Statistical Analysis

Fluorescence digital images were recorded using an LSM 510 Meta confocal microscope or an Axioskop 2 Plus fluorescence microscope (both from Carl Zeiss). In all experiments, measurements were performed at the border of SVZ neurospheres where migrating cells form a pseudo-monolayer of cells. For the self-renewal assay, the experiments were replicated in six independent culture preparations and each experimental condition was assayed in four different wells. For the remaining experiments, each condition was assayed in three different coverslips, and except where otherwise specified, the experiments were replicated in three independent culture preparations. For BrdU ELISA, each experimental condition was reproduced in 4 different wells of a 96 well plate and experiments were assayed 4 times. Background values obtained in the negative control wells were subtracted to all values. Within each experiment, the mean of values of optical density read at 450 nm in control wells were set to 100% and optical densities of experimental conditions are expressed as percentages of the Control condition. Number of neurospheres that were immunoreactive for Olig2, Olig2/GFAP or Olig2/GFAP/DCX was counted as a percentage of total neurospheres. Percentages of Sox2 cell pairs were obtained from counting about 60 cell pairs in triplicate coverslips obtained from 3 independent cultures. Percentages of BrdU, βIII tubulin/BrdU, NeuN/BrdU, NeuN, VGAT, TH/βIII tubulin and GFP/MAP2 immunoreactive cells were calculated from cell counts in five independent microscopic fields in each coverslip with a 40× objective (approximately 100 cells per field). Quantification of the number of ramifications as well as total neurite length positive for MAP2 *per* MAP2/GFP cell was performed in 3 culture preparations in approximately 20 non-overlapping fields *per* coverslip (40× magnification). Software used was Axiovision, release 4.6 (Carl Zeiss).

For SCCI experiments, the percentage of neuronal-like responding cells (with a Hist/KCl ratio below 0.8) was calculated on the basis of one microscopic field per coverslip, containing approximately 100 cells (40× magnification), in a total of at least 3 independent cultures where each conditions are triplicate.

Data are expressed as means ± *standard* error *of the mean* (SEM). Statistical significance was determined by using the unpaired two-tailed Student’s t test or one-way analysis of variance followed by Bonferroni or Dunnett’s-multiple comparison test, with p<0.05 considered to represent statistical significance.

## Results

### SVZ Cells Express CB1R

**Figure 1:**
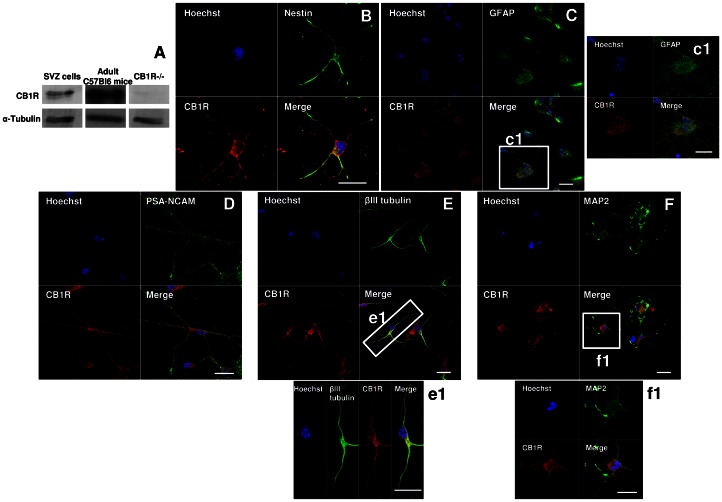
SVZ cells express CB1R. **A:** Detection of CB1R by Western blotting in SVZ. Lane 1 corresponds to SVZ proliferating cells, lane 2 to SVZ extract from adult C57Bl6 mice and lane 3 to the negative control (total proteins from CB1R-KO mice). **B–F:** Representative confocal digital images depicting CB1R immunoreactivity in SVZ cells after 7 days of differentiation [CB1R (in red); nestin (in green), GFAP (in green), PSA-NCAM (in green), DCX (in green), βIII tubulin (in green), MAP2 (in green) and Hoechst 33342 (used to visualize cell nuclei, in blue)]. c1, e1 and f1 are magnifications of squares in C, E and F, respectively. Scale bars = 20 µm. SVZ: subventricular zone; GFAP: Glial fibrillary acidic protein; PSA-NCAM: Polysialylated neural cell adhesion; βIII tubulin: Neuron-specific class III beta-tubulin; MAP2: Microtubule-associated protein 2; CB1R: CB1 receptor.

The expression of CB1R protein in SVZ-derived cells was confirmed by western blotting (Fig. 1A) and immunocytochemistry (Fig. 1B–E). In fact, CB1R was detected in immature nestin-positive SVZ cells adhered on poly-D-lysine coverslips (Fig. 1B) and in GFAP-positive astrocytes indicating that its expression is also maintained in astrocytes (Fig. 1C, c1). We have also observed the expression of CB1R in Sox2-positive single cells dissociated from the SVZ during the cell culture procedure (Fig. S2). Moreover, CB1R expression was detected in the neuronal lineage since it was observed in neurons expressing PSA-NCAM (Fig. 1D), βIII tubulin (Fig. 1E, e1) and MAP2 (Fig. 1F, f1).

### (R)-(+)-Methanandamide Promotes Self-renewal and Multipotency of SVZ Cell Cultures

**Figure 2:**
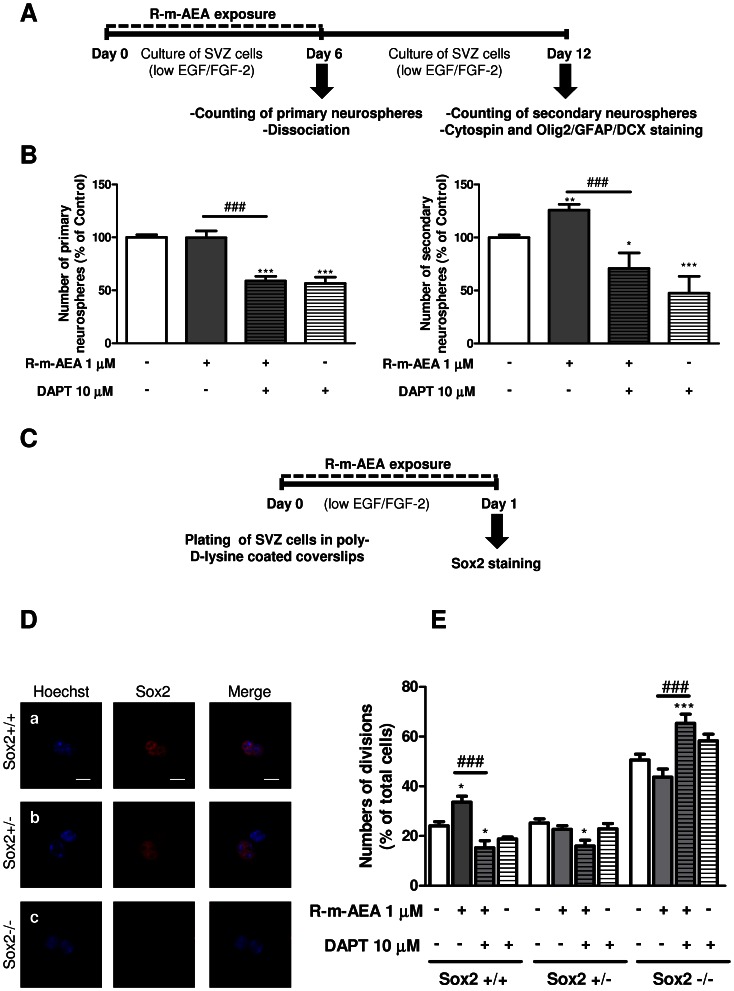
(R)-(+)-Methanandamide promotes self-renewal. **A:** Experimental protocol. **B:** Bar graphs represent the number of primary and secondary neurospheres. Data are expressed as mean ± SEM. N = 6. *P<0.05, **P<0.01 and ***P<0.001 using Dunnett’s multiple comparison test, for comparison with control; ^###^P<0.001 using Dunnett’s multiple comparison test, for comparison with R-m-AEA. **C:** Protocol used for studying cell-fate. **D:** Confocal digital images of cell pairs obtained following (a) the symmetrical division of a SVZ cell into two Sox2+ cells (Sox2+/+), (b) the asymmetrical division into a Sox2+ and a Sox2- progenitor (Sox2+/−) and (c) the symmetrical terminal division into two Sox2- progenitors (Sox2−/−). Scale bars 20 µm. **E:** Bar graph illustrates the number of each type of cell divisions counted. Data are expressed as the percentage of total cell pairs and are represented as the mean ± SEM. N = 5. *P<0.05 and ***P<0.001 using Bonferroni’s multiple comparison test, for comparison with the respective controls; ^###^P<0.001 using Bonferroni’s multiple comparison test, for comparison with the respective R-m-AEA. SOX2: sex determining region Y-box 2.

Neural stem-like cells are characterized *in vitro* by both their capacity to give rise to neurospheres and to self-renew when cultured in the presence of mitogens [28]. Exposure of freshly dissected SVZ cells to 1 µM R-m-AEA during 6 days did not affect the number of primary neurospheres generated as compared to control untreated cultures (control: 100±2.46%; R-m-AEA 1 µM: 99.67±6.37%; Fig. 2A, B). However, the number of primary neurospheres was decreased when the cells were co-treated with R-m-AEA together with an inhibitor of the Notch pathway (DAPT) (R-m-AEA 1 µM+DAPT 10 µM: 58.97±4.04%; DAPT 10 µM: 56.53±6.09%, N = 3; P<0.001). On the other hand, a higher number of secondary neurospheres was generated from SVZ cells derived from R-m-AEA treated cells in comparison with control SVZ cultures (control: 100.0±2.47%; R-m-AEA 1 µM: 125.9±5.42%; N = 9, P<0.01; Fig. 2B), thus indicating that R-m-AEA priming promoted SVZ cells capacity to self-renew. This effect is lost with co-priming with 10 µM DAPT (R-m-AEA 1 µM+DAPT 10 µM: 70.84±14.54%, N = 3, P<0.001), while the inhibitor by itself also blocked the self-renewal capacity (DAPT 10 µM: 47.58±15.83%). These data suggest that the notch pathway mediates CB1R induction of self-renewal.

The capacity of R-m-AEA to promote self-renewal of SVZ cells was further tested by plating SVZ cells for 24 h in medium complemented with R-m-AEA or control medium. After the culture session, cells were stained for Sox2, a marker of neural stem cells with the ability to self-renew (Fig. 2C). Cell pairs resulting from the division of a single SVZ stem/progenitor cell were counted and categorized in 3 groups according to their Sox2 expression: in both daughter cells (Sox2+/+), in only one of the daughter cell (Sox2+/−) and no expression (Sox2−/−) (Fig. 2D). R-m-AEA at 1 µM induced a significant increase in the percentage of Sox2+/+ cell pairs (control: 24.12±1.67%; R-m-AEA: 33.61±2.42%; N = 6, P<0.05) with a concomitant decrease of Sox2−/− cell pairs as compared with controls (control: 50.59±2.34%; R-m-AEA: 43.64±3.31%; N = 5) (Fig. 2E). These data suggest that R-m-AEA induced self-renewing divisions. Moreover, we observed that DAPT blocked the increase in Sox2+/+ cell pairs (R-m-AEA 1 µM+DAPT 10 µM: 15.23±2.85%), while promoting Sox2−/− cell pairs (R-m-AEA 1 µM+DAPT 10 µM: 65.3±3.78%), further suggesting that CB1R role in self-renewal is dependent of the notch pathway.

Moreover, we evaluated if R-m-AEA exposure altered the multipotency of the formed neurospheres, by adhering secondary free-floating neurospheres to glass slides and analysing the expression of Olig2, GFAP and DCX which are markers of oligodendrocyte progenitors, astrocytes and neurons, respectively. We observed an increased number of tripotent neurospheres expressing simultaneously Olig2, GFAP and DCX (Control: 78.63±2.95%; R-m-AEA 1 µM: 87.63±2.84%; P<0.01, Fig. S3), whereas the number of bipotent neurospheres expressing Olig2 and GFAP was significantly reduced (Control: 20.67±1.89%; R-m-AEA 1 µM: 11.71±2.66%; N = 3, P<0.05, Fig. S3).

### (*R*)-(+)-Methanandamide Stimulates Cell Proliferation

**Figure 3:**
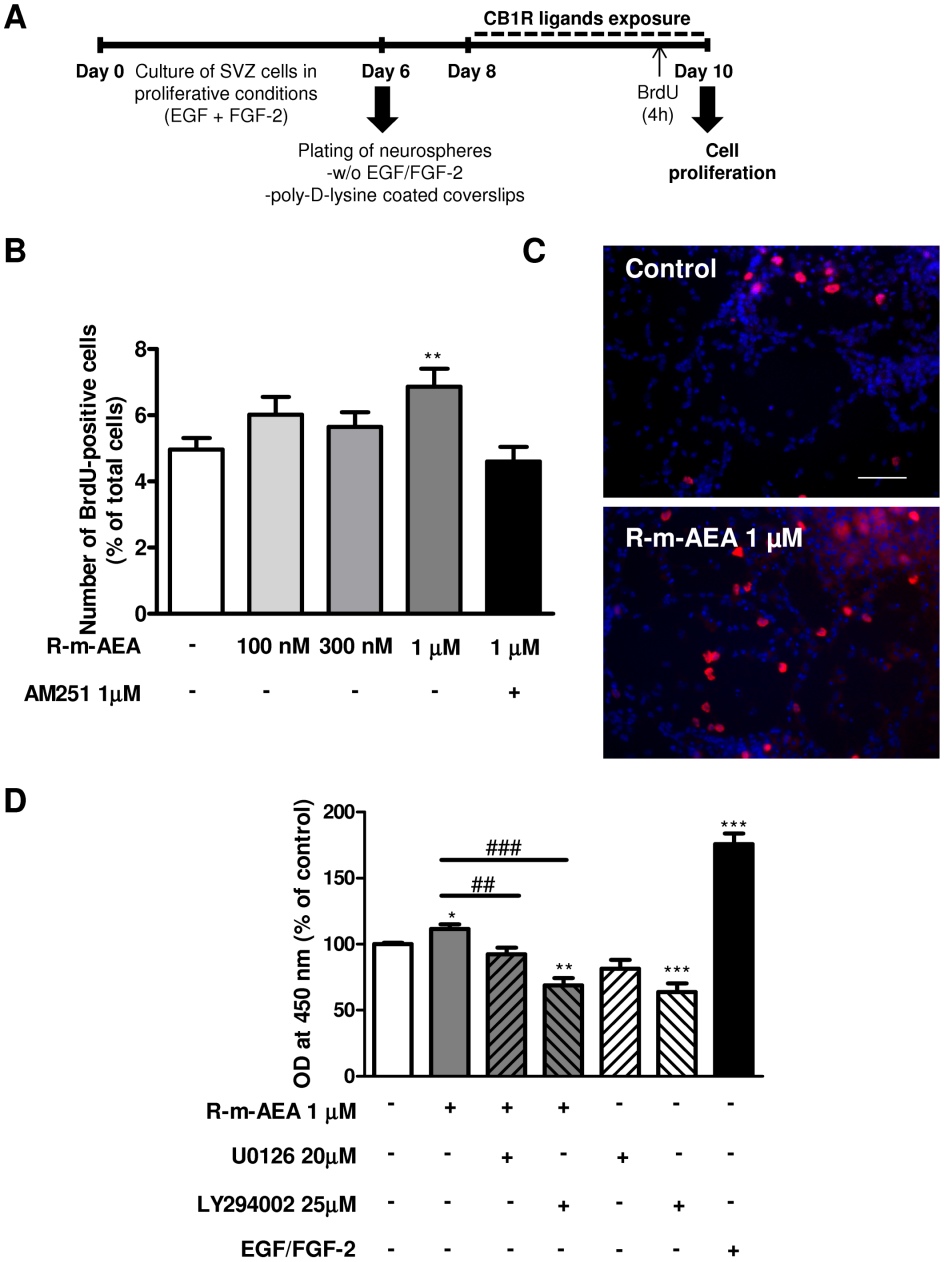
(R)-(+)-Methanandamide induces cell proliferation in SVZ cell cultures. **A:** Protocol used for studying cell proliferation. **B:** Bar graph depicts the numbers of BrdU-positive cells expressed as percentage of the total number of cells. Data are expressed as mean ± SEM. N = 3–5. **P<0.01 using Dunnett’s multiple comparison test, for comparison with control. **C:** Representative digital images of BrdU (red nuclei)-positive cells and Hoechst 33342 staining (blue nuclei) in SVZ cultures. **D:** Amount of BrdU incorporation evaluated by densitometry (at 450 nm). N = 10. *P<0.05, **P<0.01 and ***P<0.001 using Dunnnet’s multiple comparison test, for comparison with control; ^##^P<0.01 and ^###^P<0.001 using Dunnnet’s multiple comparison test, for comparison with R-m-AEA; Scale bar = 50 µm.

To determine whether R-m-AEA modulates cell proliferation, increasing concentrations of R-m-AEA (100 nM, 300 nM, 1 µM) were applied on SVZ cells in SFM devoid of growth factors, for 48 h (Fig. 3A). BrdU, an analog of the thymidine nucleotide, was added during the last 4 h of the culture to label cells that went through S-phase. After fixation, incorporated BrdU was immunolabeled and positive nuclei were counted. A significant increase in the number of BrdU-immunopositive nuclei was obtained in cultures incubated with 1 µM but not with 100 nM or 300 nM, when compared with control (control: 4.97±0.35%; R-m-AEA 1 µM: 6.87±0.541%; R-m-AEA 100 nM: 6.01±0.54%; R-m-AEA 300 nM: 5.65±0.44%; N = 3–5, P<0.01) (Fig. 3B). Moreover, the effect caused by R-m-AEA was blocked by the presence of the CB1R antagonist AM 251 [R-m-AEA+AM 251∶4.6±0.44%)], further indicating that R-m-AEA effect on cell proliferation is CB1R-mediated (Fig. 3B). Furthermore, the effect of R-m-AEA or AM 251 on cell viability was evaluated after 48 h of drug treatment. Apoptotic nuclei were stained by the TUNEL (Terminal deoxynucleotidyl transferase dUTP nick end labeling) method and no significant differences in the number of TUNEL-positive nuclei were found, indicating that none of the drugs were toxic to the cells (control: 11.38±1.14%; R-m-AEA 100 nM: 13.12±2.04%; R-m-AEA 300 nM: 10.97±1.07%; R-m-AEA 1 µM: 13.08±2.33%; AM 251 1 µM: 13.3±0.76%; as a control, culture medium containing 0.20% Ethanol: 12.67±0.71%; N = 3, data not shown).

ERK1/2 is a mitogen-activated protein kinase (MAPK) known to mediate proliferation of neuronal progenitor cells [32]. Moreover, it has been shown that PI3K/AKT pathway is also involved in SVZ cell proliferation [33]–[35]. To investigate whether the increase in cell proliferation mediated by R-m-AEA treatment is dependent on ERK and/or PI3K/AKT pathways, we evaluated proliferation by quantifying BrdU incorporation with an ELISA detection method and used selective inhibitors of the signaling pathways.

The increase in proliferation mediated by R-m-AEA was blocked when cells were co-incubated with 20 µM U0126 a MAPK kinase 1/2 inhibitor (MEK1/2) or with 25 µM LY294002, a PI3K inhibitor, together with R-m-AEA (Control: 100.00±1.06%; 1 µM R-m-AEA: 111.5±3.66%; 1 µM R-m-AEA +20 µM U0126∶92.25±5.09%; 1 µM R-m-AEA +25 µM LY294002∶68.73±5.72%; N = 10) (Fig. 3D). These results suggested a pro-proliferative action of CB1R activation involving MAPK/ERK and AKT signaling pathways.

To investigate whether R-m-AEA activates the ERK1/2 signaling pathway, SVZ cell cultures were exposed to 1 µM R-m-AEA for 15 min and 30 min and cells were processed for western blotting to detect phosphorylated activated form of ERK1/2 (P-ERK1/2). We observed that P-ERK1/2 protein levels increased to 138.4±12.12% (N = 5, P<0.05) in cultures exposed for 15 min to R-m-AEA when comparing with control condition (set to 100%) (Fig. S4).

### (*R*)-(+)-Methanandamide Induces Neuronal Differentiation *via* CB1R Activation

**Figure 4:**
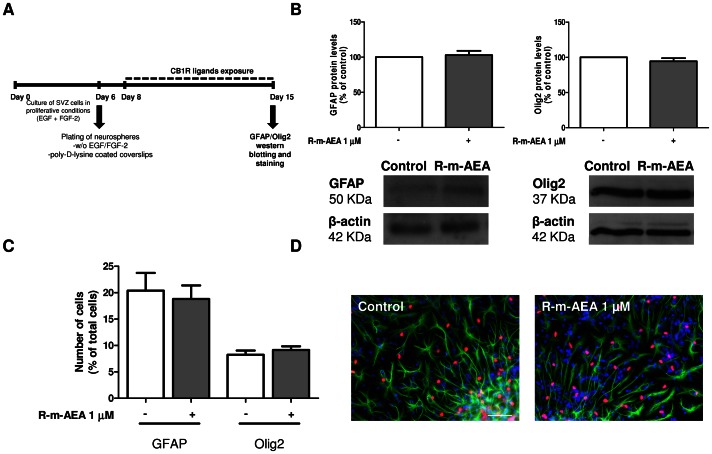
(R)-(+)-Methanandamide does not induce glial differentiation in SVZ cultures through CB1R activation. **A:** Protocol used for studying glial differentiation. **B:** Western blot analysis of GFAP and Olig2 protein levels in SVZ. Data are expressed as mean ± SEM. N = 4. **C:** Bar graph depicts the number of GFAP and Olig2-positive cells, expressed as the percentage of total cells *per* culture. Data are expressed as mean ± SEM. N = 3. **D:** Representative fluorescent digital images of GFAP-positive cells (green), Olig2-positive cells (red) and Hoechst staining (blue nuclei). Scale bar = 50 µm.

The effects of R-m-AEA on glial cell differentiation were assessed by western blotting from SVZ cells treated with R-m-AEA for 7 days. Quantification of GFAP and Olig2 protein levels was performed and we found no effect of R-m-AEA treatment (GFAP: control set at 100%, R-m-AEA 1 µM: 102.9±5.90%; Olig2: control set at 100%, R-m-AEA 1 µM: 94.28±4.57%, N = 4), indicating that R-m-AEA does not affect glial differentiation (Fig. 4B). Moreover, we performed immunocytochemistry against GFAP and Olig2 in SVZ cultures incubated for 7 days in the absence (control) or presence of 1 µM R-m-AEA. No differences in the numbers of astrocytes and oligodendrocytes were observed between the conditions (GFAP, control: 20.38±0.98%, R-m-AEA: 18.83±0.81%; Olig-2, control: 8.25±0.8%, R-m-AEA: 9.15±0.69%; N = 3) (Fig. 4C,D).

**Figure 5:**
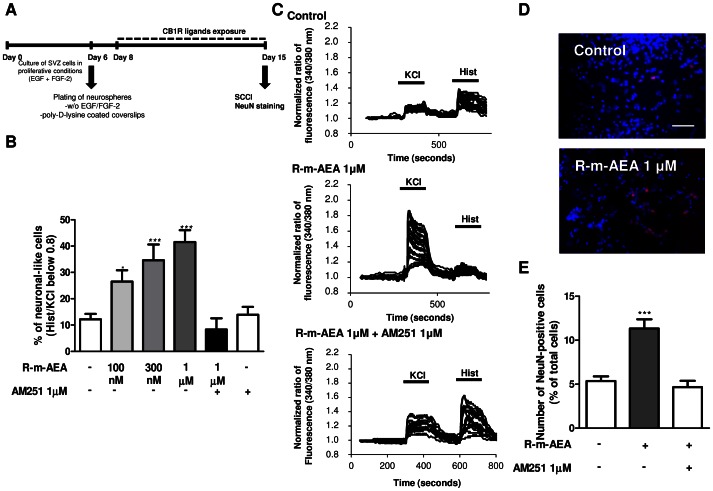
(R)-(+)-Methanandamide induces neuronal differentiation in SVZ cultures through CB1R activation. **A:** Schematic representation of the protocol. **B:** Bar graph depicts the number of neuronal-like responding cells expressed as percentages of total cells analyzed by SCCI. N = 4–8. *P<0.05, ***P<0.01 using Dunnett’s multiple comparison test, for comparison with control. **C:** Representative SCCI profiles of response of about 20 cells in control, in R-m-AEA and in R-m-AEA+AM 251 treated cultures. **D**: Representative fluorescent digital images of NeuN-positive neurons (red) and Hoechst staining (blue nuclei). Scale bar = 50 µm. **E:** Bar graph depicts the number of NeuN-positive cells, expressed as the percentage of total cells *per* culture. Data are expressed as mean ± SEM. N = 5 ***P<0.001 using Dunnett’s multiple comparison test, for comparison with control. SCCI: single cell calcium imaging; NeuN: Neuronal Nuclei.

**Figure 6:**
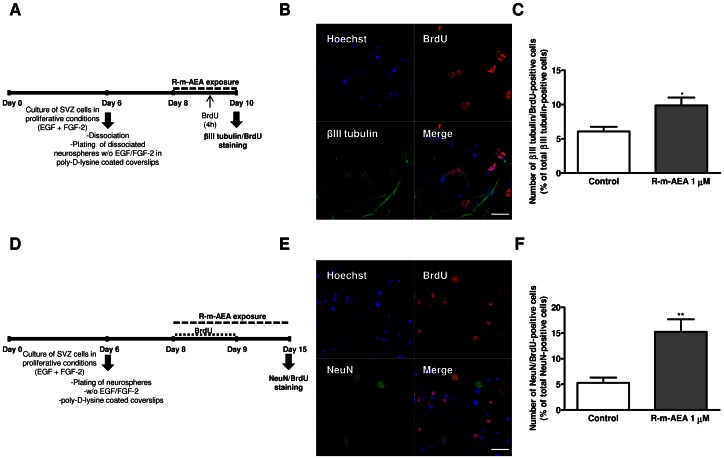
(R)-(+)-Methanandamide promotes proliferation of neuroblasts. **A, D:** Experimental protocol. **B, E:** Representative confocal digital images of BrdU (red), βIII tubulin (green) and Hoechst staining (blue) (B) and of BrdU (red), NeuN (green) and Hoechst staining (blue) (E). Scale bar = 20 µm. **C, F:** Bar graphs depict the number of βIII tubulin/BrdU-positive cells expressed as percentage of total βIII tubulin positive cells (C) and NeuN/BrdU-positive cells expressed as percentage of total NeuN-positive cells (F) per culture. Data are expressed as mean ± SEM. N = 3. *P<0.05, **P<0.01 using unpaired Student’s t test for comparison with control. βIII tubulin: Neuron-specific class III beta-tubulin; NeuN: Neuronal Nuclei.

We next investigated whether R-m-AEA affected neuronal differentiation. For that purpose, SVZ cells were treated with R-m-AEA for 7 days (Fig. 5A). To functionally evaluate neuronal differentiation in SVZ cultures, we used a method previously described by us [26], [31], [36]. For that we analysed the variations of [Ca^2+^]_i_ at single cell level upon KCl and histamine (Hist) stimulations, Hist/KCl ratios below 0.8 being characteristic of SVZ-derived neuronal-like cells [31]. In control cultures we observed a predominant immature-like profile, characterized by an increase in [Ca^2+^]_i_ in response to histamine but a small response or no response to KCl stimulation (Fig. 5C). Interestingly, the majority of R-m-AEA treated SVZ cells displayed an increase in [Ca^2+^]_i_ in response to KCl but not to histamine stimulation, consistent with a neuronal-like profile (Fig. 5C). Quantification of the percentage of cells displaying a Hist/KCl ratio below 0.8 showed that R-m-AEA is proneurogenic (Control: 12.18±2.09%; R-m-AEA 100 nM: 26.54±4.28%, P<0.05; R-m-AEA 300 nM: 34.58±6.0%, P<0.001; R-m-AEA 1 µM: 41.52±4.49%, P<0.001; N = 4–8; Fig. 5B). Moreover, we observed that the CB1R antagonist AM 251 (1 µM) was capable of blocking R-m-AEA-induced neuronal differentiation [R-m-AEA 1 µM+AM 251 1 µM: 8.29±4.27%; AM 251 1 µM: 13.88±3.00%; N = 4, Fig. 5B)]. This method allowed us to quantify glial cells as these cells do not respond to KCl nor to histamine and thereby display a ratio of response close to 1 [31]. No differences were obtained between the conditions (Fig. S5A) further attesting that CB1R stimulation does not affect glial differentiation (ratio Hist/KCl 0.9–1) (Control: 38.37±3.45%: R-m-AEA 100 nM: 35.95±3.47%; R-m-AEA 300 nM: 29.68±3.70%; R-m-AEA 1 µM: 30.75±4.61%, N = 8). Moreover, we also observed that CB1R activation promoted a decrease in the % of immature-like cells (ratio Hist/KCl 1–1.3) (Control: 26.22±3.28%; R-m-AEA 100 nM: 10.52±2.39%; R-m-AEA 300 nM: 11.82±3.03%; R-m-AEA 1 µM: 9.49±1.50%, N = 8) (Fig. S5B). Further supporting the role of CB1R activation in neuronal differentiation, R-m-AEA induced an increase in NeuN-positive cells when compared with control cultures (Control: 5.35±0.53%; R-m-AEA 1 µM: 11.33±1.05%; N = 4–5, P<0.001), and CB1R antagonist blocked this effect (R-m-AEA 1 µM+AM 251 1 µM: 4.66±0.73%) (Fig. 5D, E), further suggesting that R-m-AEA promotes neuronal differentiation. Additionally, we also found that the treatment with R-m-AEA increased proliferation of neuronal progenitors, as observed by the increase in βIII tubulin/BrdU-positive cells in SVZ cultures treated with R-m-AEA for 48 h (Control: 6.01±0.68%; 1 µM R-m-AEA: 9.87±1.16%; N = 3, P<0.05) (Fig. 6A,B,C), without affecting the total number of βIII tubulin-positive cells (Control: 27.0±1.39%; 1 µM R-m-AEA: 26.15±2.01%; N = 3, data not shown). Using a pulse of BrdU together with R-m-AEA for the first 24 h of treatment followed by a chase of 6 days (without BrdU) in the absence (control) or in the presence of R-m-AEA (Fig. 6D) we observed an increase in the number of NeuN/BrdU-positive cells in the treated condition as compared to control, indicating that R-m-AEA may interfere in the early stages of neuronal differentiation, committing progenitors towards a neuronal fate (Control: 5.31±0.99%; 1 µM R-m-AEA: 15.24±2.43%; N = 3, P<0.01) (Fig. 6 E, F).

**Figure 7:**
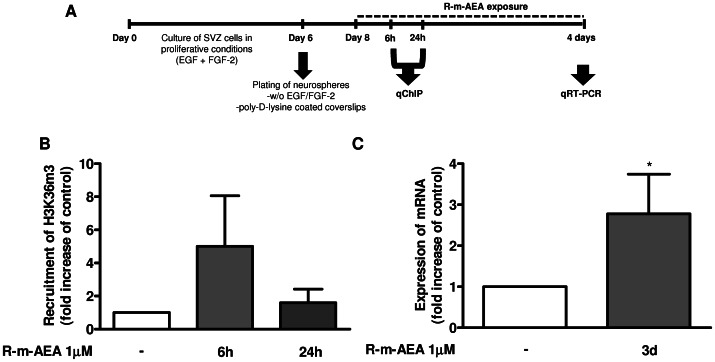
(R)-(+)-Methanandamide promotes the expression of the proneurogenic genes *Ngn1*. **A:** Scheme of the protocol. **B:** Bar graph depicts the fold increase of H3K36m3 recruitment in the promoter region of *Ngn1* gene quantified by qChIP analysis. **C**: Bar graph depicts the fold increase of mRNA expression for Ngn1 protein evaluated by qRT-PCR analysis. Data are expressed as mean ± SEM. N = 4–7. *P<0.05, using Dunnett’s test for comparison with control (set to 1). H3K36m3: Histone H3 trimethylated on lysine 36; Ngn1: Neurogenin 1; qChIP: quantitative chromatin immunoprecipitation; qRT-PCR: quantitative real time polymerase chain reaction.

To disclose if R-m-AEA has proneurogenic effects through histone modifications associated with the transcription of the proneurogenic gene *Ngn1*, we performed qChIP targeting the trimethylated lysine 36 of the H3 histone (H3K36m3) followed by a qPCR analysis (Fig. 7A). This specific histone methylation is associated with transcription-active euchromatin [37], therefore lineage potential can be studied by observing histone methylation associated with the transcription of a proneurogenic gene. We observed an early increased recruitment of H3K36m3 to the regulatory elements of *Ngn1* in SVZ cells exposed to 1 µM (*R*)-(+)-Methanandamide, for 6 h (5.00±3.06; control set to 1, N = 3), suggesting the enhancement of the transcriptional activity of *Ngn1* (Fig. 7B). Using qRT-PCR analysis, we confirmed that 1 µM R-m-AEA triggered a significant increase in *Ngn1* mRNA levels (2.77±0.697 fold increase; N = 4, P<0.05) in SVZ cells treated for 3 days as compared with controls (set to 1) (Fig. 7C).

**Figure 8:**
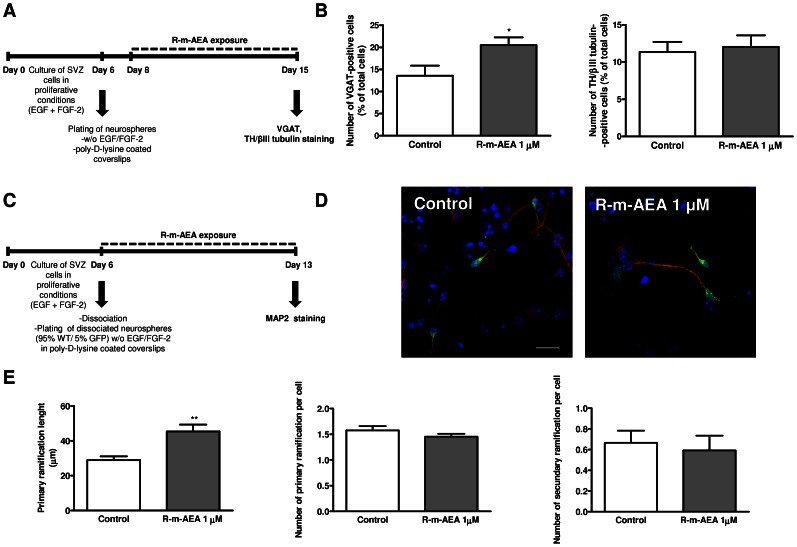
(R)-(+)-Methanandamide induces the differentiation of GABAergic neurons and neuritogenesis. **A:** Schematic representation of the protocol. **B:** Bar graph depicts the numbers of either VGAT- or TH/βIII tubulin-positive cells, expressed as percentage of total cells. The data are expressed as percentage ± SEM. N = 3. *P<0.05 using unpaired Student’s t test for comparison with control. **C:** Schematic representation of the protocol used for studying neuritogenesis. **D:** Representative confocal digital images of the GFP (green), MAP2 (red), Hoechst staining (blue), in control cultures and in cultures exposed to R-m-AEA. Scale bar = 20 µm. **E:** Bar graphs depict (from left to right): total length (µm), number of primary and number of secondary ramifications of MAP2 neurites per cell. N = 3. **P<0.01 using unpaired student’s t test for comparison with control. MAP2: Microtubule-associated protein 2; TH: tyrosine hydroxylase; βIII tubulin: Neuron-specific class III beta-tubulin; VGAT: vesicular GABA transporter.

We then investigated whether 1 µM R-m-AEA influences the phenotype of the neuronal cells produced in the cultures. For that purpose immunolabelings for the vesicular GABA transporter (VGAT) and the tyrosine hydroxylase (TH) were performed on 7 day-treated cultures since GABAergic and dopaminergic neurons are the two major phenotypes of SVZ-derived neurons (Fig. 8A). We observed an increase in VGAT-positive cells (Control: 13.57±2.29%; R-m-AEA: 20.52±1.76%; N = 3, P<0.05), while, in the same experimental conditions, we did not observed an increase in TH-positive cells (Control: 11.34±1.38%; R-m-AEA: 12.04±1.55%) (Fig. 8B). Finally, consistently with the observed proneurogenic action of endocannabinoids, we investigated whether the R-m-AEA triggered the growth of neurites. For that we exposed mixed cultures of WT and GFP mice for 7 days to 1 µM R-m-AEA and we evaluated the length and the number of primary and secondary ramifications of MAP2 positive neurites (Fig. 8C, D). Exposure of SVZ cells to R-m-AEA induced a marked increase in the length of MAP2/GFP-positive neurites per cell (Control: 29.0±2.14 µm; 1 µM R-m-AEA: 45.41±3.96 µm; N = 3, P<0.01) (Fig. 8E). However, the number of primary and secondary ramifications of MAP2/GFP-positive treated cells was similar to control cultures (Fig. 8E).

## Discussion

The role of endocannabinoids in the regulation of neurogenesis has been the subject of several recent studies; however most of these studies address the effects of endocannabinoids on hippocampal neurogenesis with emphasis on proliferation. Here we examined the effects of endocannabinoids on SVZ-derived cells, given that SVZ represents the main stem cell reservoir in the rodent adult brain. These cells may be used for cell replacement therapies if efficient proneurogenic compounds are identified. In our study, we disclosed the modulatory effects of endocannabinoids on SVZ proliferation, cell fate decision, survival and differentiation.

Firstly, we observed by western blotting that CB1R is expressed in SVZ cell cultures. CB1R is indeed expressed in immature nestin-positive cells (marker of stem-like cells *in vitro*), in astrocytes (GFAP-positive cells) and in immature neurons (PSA-NCAM- and DCX-positive cells). Others have shown that neural progenitor cells express CB1R, in fact CB1R was found to be expressed *in vivo* in the SVZ and DG and in nestin-positive cells from neural progenitor cultures [17], [20], [21], [38].

We have also found that R-m-AEA promotes self-renewal and multipotency of SVZ cell cultures, as observed by an increase in the total number of secondary neurospheres that were also tripotent (Olig2/GFAP/DCX). Moreover, CB1R agonist treatment induced self-renewing divisions, resulting in an increased number of Sox2+/+ pairs of daughter cells. Accordingly, these data indicate that CB1R activation promotes stemness in SVZ cells. To our knowledge, this is the first time that such a property for CB1R activation is directly demonstrated, although it was already suggested that CB1R activation might be important to maintain self-renewal of embryonic cortical neural stem cells cultures in proliferation conditions [39]. Recently, Compagnucci and collaborators [40] have shown by gene array profiling that in embryonic mice cortical neural stem cells under differentiation conditions (no growth factor, 1% serum and upon adherence), CB1R activation increases the expression of genes involved in neuronal differentiation while it decreased the genes involved in stemness. However, we herein studied the role of CB1R in stemnes in the postnatal neural stem cells under proliferation conditions. In concordance with their results the number of immature cells in our cultures, evaluated according to their response to histamine but not to KCl [31], decreased with R-m-AEA treatment under differentiation conditions. In addition, we observed that inhibiting the Notch pathway with DAPT blocked the increase in self-renewal induced by R-m-AEA treatment, suggesting that the notch pathway mediates CB1R induction of self-renewal. In fact, Tanveer and collaborators [41] have shown that anandamide, increases Notch-1 signaling in cortical neurons exposed to amyloid-beta and in the cortex of aged rats.

Moreover, we observed an increase in the number of BrdU-positive cells in cultures treated with R-m-AEA, and this effect was blocked by a CB1R selective antagonist, further showing that R-m-AEA effect is CB1R-mediated. In fact, there is an emerging consensus that endocannabinoid signaling plays a major role in proliferation. In this context, reduction in neural stem cell proliferation was seen in both the hippocampus and SVZ in CB1R-KO animals [21], [22], [38] and in diacylglycerol lipase (DAGL, the enzyme that synthesize 2-AG, one of the main endocannabinoids in the CNS)-KO mice [18]. Accordingly, knocking-out or inhibiting the enzyme fatty acid amide hydrolase (FAAH, an enzyme involved in the breakdown of endocannabinoids), promotes the increase in proliferation [17], [38]. Furthermore, several data also suggest that CB1R activation induces proliferation [17], [20], [24]. However, other reports also suggest that CB2R are expressed by neural stem cells, and that CB2R-selective agonists and antagonists modulate the generation of cultured neural stem cell and precursor cell proliferation [19], [42]–[44]. We found that incubation of SVZ stem/progenitor cell cultures with R-m-AEA together with U0126 (an inhibitor of ERK pathway) or with LY294002 (an inhibitor of PI3K pathway) promotes a decrease in BrdU incorporation, when comparing with CB1R agonist treatment alone, demonstrating that these pathways (ERK and PI3K) play pivotal roles in the CB1R-dependent modulation of proliferation.

In fact, others have already shown that ERK and PI3K pathways are important regulators of CB1R-mediated proliferation [13], [20], [45]. Most of the studies claiming that CB1R activation promotes neurogenesis addressed their role in proliferation rather than in cell differentiation. Therefore, we studied the effect of R-m-AEA treatment, in differentiation conditions, on glial and neuronal differentiation. We observed that CB1R activation did not induce differentiation of GFAP-positive astrocytes or Olig2-positive oligodendrocytes. However, previous published results are apparently discordant because some authors have shown that endocannabinoid treatment promotes astroglial and/or oligodendroglial differentiation [38], [41], [46], [47] while others have shown that CB1R activation promotes neuronal differentiation [40], [48]. Our results are in accordance with an effect mediated by CB1R activation on neuronal differentiation. In fact, we found that R-m-AEA induces functional neuronal differentiation in SVZ cell cultures *via* CB1R activation and these results were further supported by an increase in the number of NeuN-positive cells. We also found that treatment with R-m-AEA increased proliferation of neuronal progenitors (βIII tubulin/BrdU-positive cells) in 48h treated cultures. Interestingly, the total number of βIII tubulin-positive cells was similar between the control and R-m-AEA treated condition, for 48 h. However after 7 days of treatment, the proportion of NeuN-positive cells was increased. R-m-AEA may increase neuronal differentiation *via* promoting proliferation of neuronal precursor cells, but also by accelerating neuronal maturation. Indeed, more progenitor cells labeled with BrdU, for the first 24 h of the culture session, ultimately differentiated into NeuN-positive mature neurons following 7 days of treatment with R-m-AEA. Moreover, we showed that GABAergic differentiation is favored by R-m-AEA treatment. Soltys and collaborators have also observed an increase in neuronal differentiation of neural progenitor cells by anadamide treatment [48]. However, Rueda and collaborators [49] showed that endocannabinoids and the CB1R agonist HU210 inhibited neuronal progenitor cell differentiation through a reduction of the ERK pathway activation in cultured embryonic cortical cells, human neural stem/progenitor cells and PC12 cells stably transfected with human CB1R. In addition, endocannabinoid signaling has been shown to promote astroglial differentiation of cortical neural progenitor cells and of adult hippocampal progenitors [38]. The divergent effects shown in the bibliography concerning the role of endocannabinoids on neurogenesis may partly rely on the use of different pharmacologic approaches. In fact, the cannabinoids may have different affinity for CB1R, CB2R, and also Transient Receptor Potential Vanilloid receptor 1 (TRPV1) [50], which may account for the observed differences. Moreover, these discrepancies can also be explained by differences in the study design, compounds used and gender of the animals [22]. Besides, the readout parameters for adult neurogenesis vary between studies with some reports focusing only on the effects of CB1R on proliferation [20], [21].

Emerging information indicates that epigenetic alterations comprising histone modifications and chromatin remodeling may be inherent to the maintenance and differentiation of neural stem cells. However, their involvement has not yet been completely understood. Recent studies indicate that lineage control genes, such as *Ngn1*, are epigenetically modified with a unique combination of histone modifications that prime them for potential activation upon cell lineage induction and differentiation [51]. Here we investigated whether R-m-AEA could promote H3K36m3 modification in the promoter region of *Ngn1*, since this is considered to be a proneurogenic gene [52]. Accordingly, upon R-m-AEA treatment, we observed an increase of H3K36m3 on *Ngn1* promoter region, which ultimately lead to an increased mRNA expression of this proneurogenic gene. Importantly, recent reports have shown that *Ngn1* is able to commit pluripotent embryonic carcinoma P19 cells to adopt a neural cell phenotype [53], [54]. Moreover, we have recently shown that histamine promotes neurogenesis and that this proneurogenic effect involves epigenetic modifications and increased expression of *Mash1*, *Dlx2*, and *Ngn1* genes [55].

The differentiation process in neurons is a complex phenomenon involving multiple changes in electrophysiological characteristics as well as changes in morphology characterized by neurite (dendritic and axonal) outgrowths. Regulation of neurite outgrowth is tightly controlled and many neurotransmitters are involved in this process [56]. Therefore, we studied neuritogenesis and we observed that primary ramification length was significantly higher in R-m-AEA-treated cultures, showing that CB1R promotes neuronal maturation. Also, Jordan and collaborators have shown that CB1R activation induces neurite outgrowth in Neuro-2A cells [56]. Moreover, recent findings in both mammals [57] and non-mammalian vertebrates [58] suggest that CB1R activation is required for axonal elongation and fasciculation. In addition, Mulder and collaborators [59] have shown that CB1R activation drives neural progenitors proliferation and migration and that in immature pyramidal cells, CB1R activation is required for axonal polarization and for the formation of long-range glutamatergic axons. More recently, it was shown that CB1R activation promotes neuronal maturation from embryonic neural stem cells in differentiation conditions [40].

Taken together, our results further dissect the role of CB1R on SVZ neurogenesis and demonstrate that its activation promotes self-renewal, proliferation, neuronal differentiation and maturation.

## Supporting Information

Figure S1 **Neurospheres are composed by stem/progenitor cells.** Representative confocal digital image depicting Sox2 and Nestin immunoreactivity in a SVZ neurosphere [Sox2 (in red), nestin (in green) Hoechst 33342 (used to visualize cell nuclei, in blue)]. Scale bar = 20 µm
(TIF)

Figure S2 **CB1R is expressed in stem/progenitor cells.** Representative confocal digital image depicting Sox2 and CB1R immunoreactivity in SVZ cells plated for 24h after culture procedure. Scale bar = 20 µm
(TIF)

Figure S3 **(R)-(+)-Methanandamide promotes multipotency.** Bar graphs depict the number of secondary neurospheres expressing either Olig2, Olig2/GFAP or Olig2/GFAP/DCX. Numbers are expressed as percentage of total spheres counted. N = 3. *P<0.05 and **P<0.01 using Bonferroni’s multiple comparison test, for comparison with the respective controls. Olig2: Oligodendrocyte transcription factor 2; GFAP: Glial fibrillary acidic protein; DCX: Doublecortin.
(TIF)

Figure S4 **(R)-(+)-Methanandamide activates ERK pathway.** Graph depicts the percentages relative to control of P-ERK1/2 protein levels normalized to total ERK1/2 in SVZ cultures. Below the graph, a representative Western blot for 44/42 kDa P-ERK and ERK is shown. N = 5. *P<0.05 using Bonferroni’s multiple comparison test for comparison with the respective controls
(TIF)

Figure S5 **(R)-(+)-Methanandamide does not induce glial differentiation in SVZ cultures through CB1R activation.** **A:** Bar graph depicts the number of glial-like (A) and immature-like (B) responding cells expressed as percentages of total cells analyzed by SCCI. N = 8. **P<0.01 and ***P<0.01 using Dunnett’s multiple comparison test, for comparison with control.
(TIF)

Methods S1 **Detailed description of methods for: a) SVZ cell cultures; b) single-cell calcium imaging (SCCI); c) quantitative chromatin immunoprecipitation (qChIP); d) cDNA synthesis and realtime RT-PCR analysis (*Ngn1*).**
(DOC)
