# Supplementary figures and images for: Activation of Type 1 Cannabinoid Receptor (CB1R) Promotes Neurogenesis in Murine Subventricular Zone Cell Cultures
Source: PLoS One. 2013 May 21;8(5):e63529. doi: 10.1371/journal.pone.0063529 (PMC3660454; doi:10.1371/journal.pone.0063529)

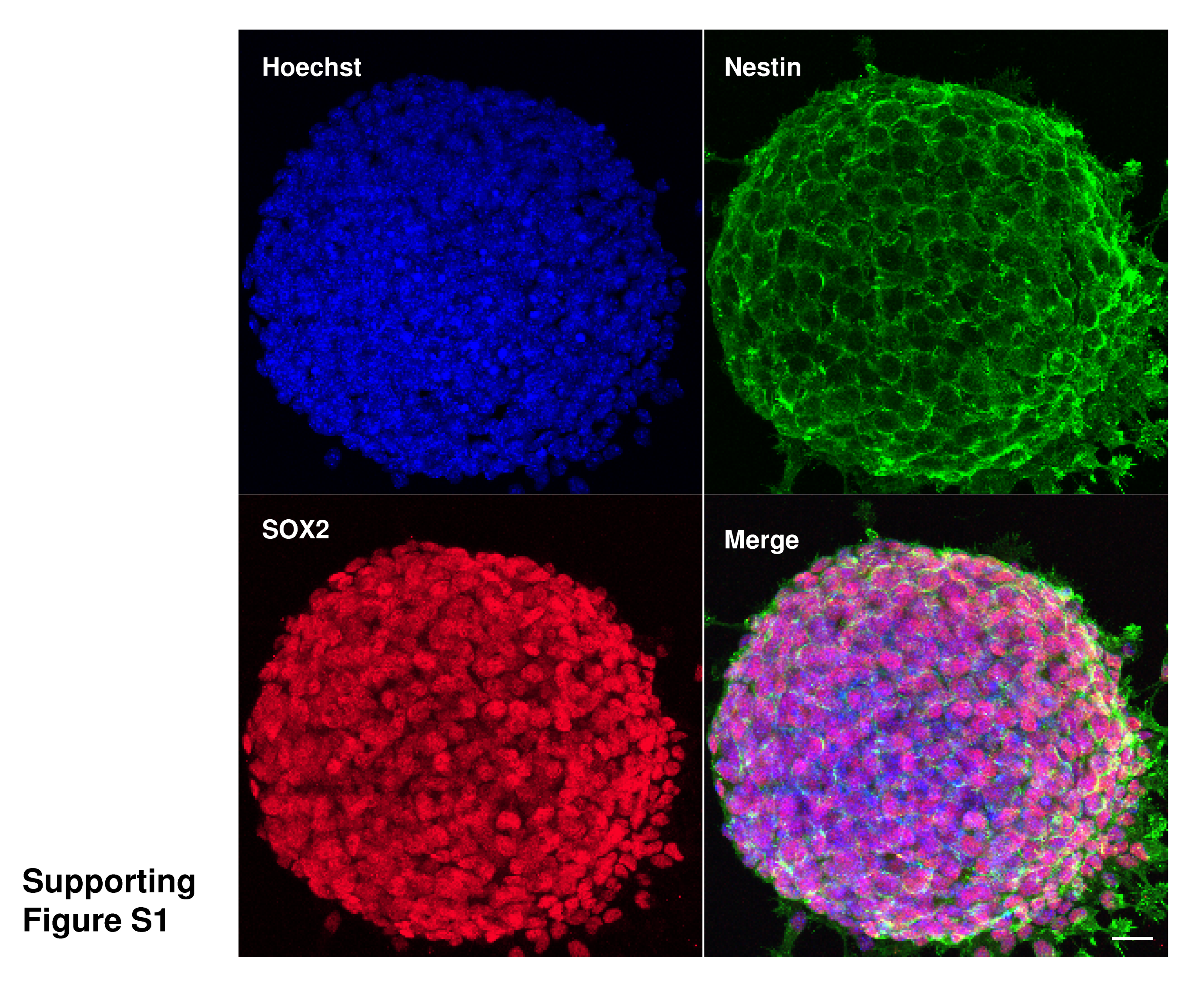

Supplement: Figure S1 — Neurospheres are composed by stem/progenitor cells. Representative confocal digital image depicting Sox2 and Nestin immunoreactivity in a SVZ neurosphere [Sox2 (in red), nestin (in green) Hoechst 33342 (used to visualize cell nuclei, in blue)]. Scale bar = 20 µm (TIF) [file pone.0063529.s001.tif]

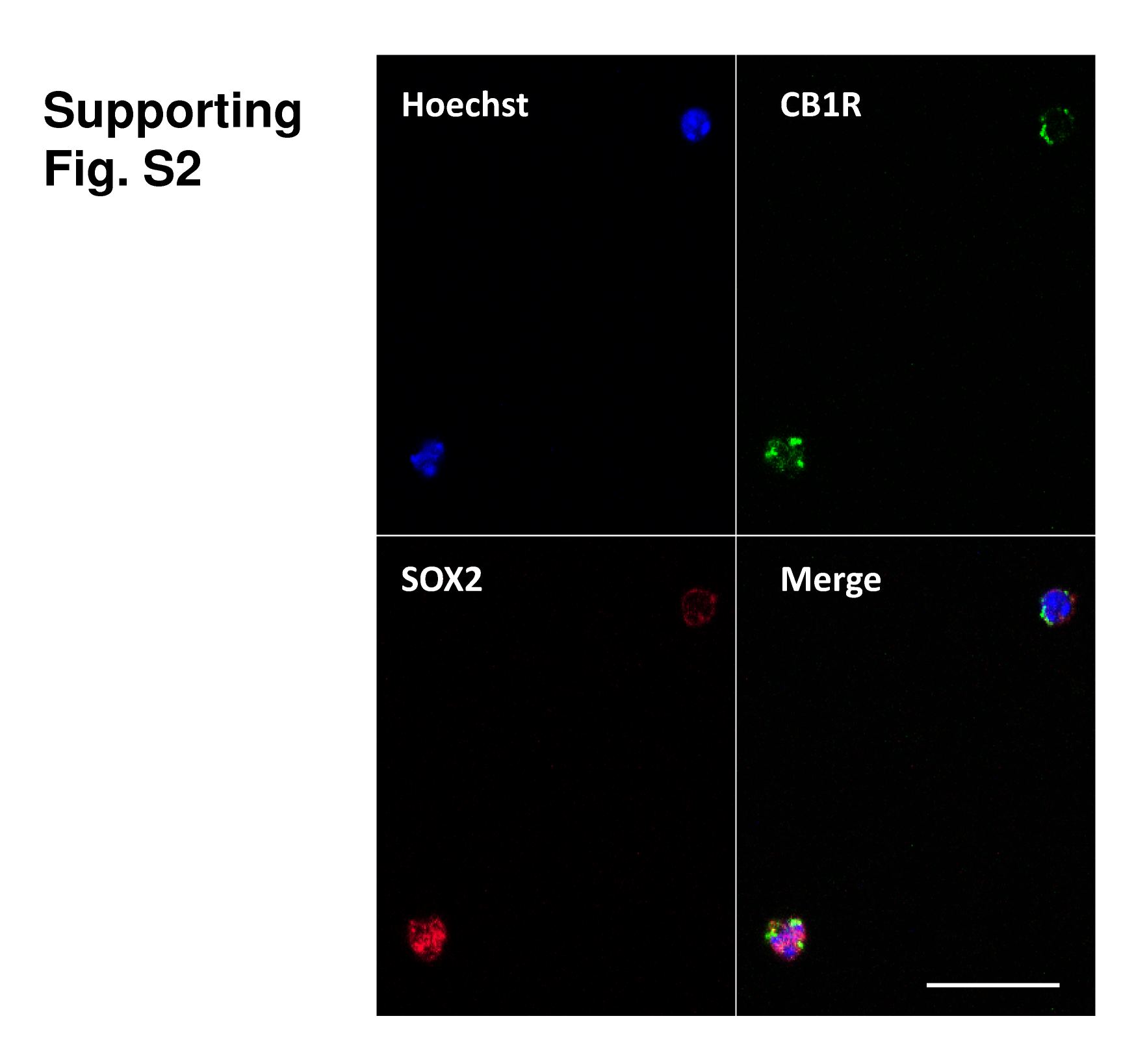

Supplement: Figure S2 — CB1R is expressed in stem/progenitor cells. Representative confocal digital image depicting Sox2 and CB1R immunoreactivity in SVZ cells plated for 24h after culture procedure. Scale bar = 20 µm (TIF) [file pone.0063529.s002.tif]

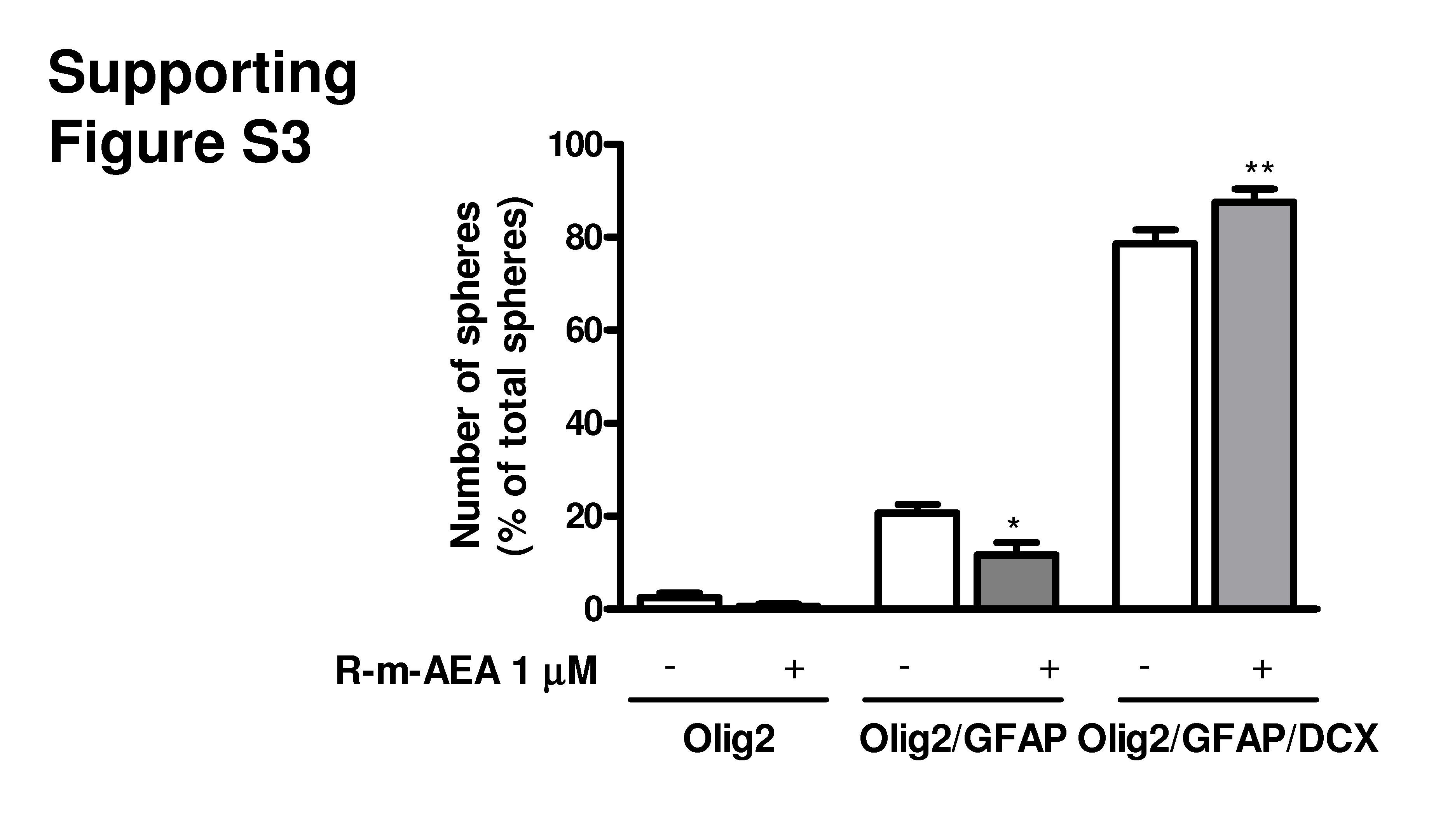

Supplement: Figure S3 — (R)-(+)-Methanandamide promotes multipotency. Bar graphs depict the number of secondary neurospheres expressing either Olig2, Olig2/GFAP or Olig2/GFAP/DCX. Numbers are expressed as percentage of total spheres counted. N = 3. *P<0.05 and **P<0.01 using Bonferroni’s multiple comparison test, for comparison with the respective controls. Olig2: Oligodendrocyte transcription factor 2; GFAP: Glial fibrillary acidic protein; DCX: Doublecortin. (TIF) [file pone.0063529.s003.tif]

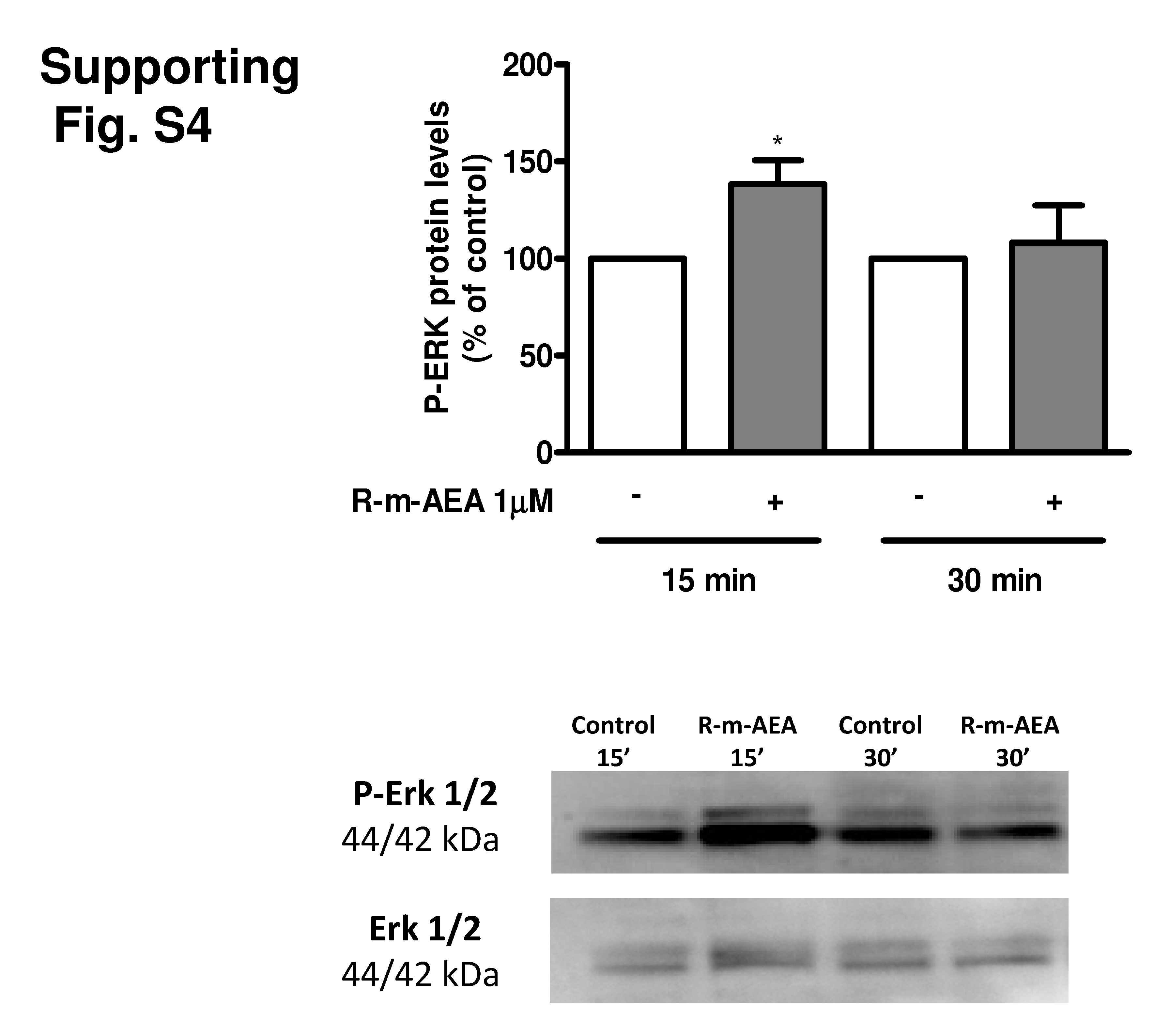

Supplement: Figure S4 — (R)-(+)-Methanandamide activates ERK pathway. Graph depicts the percentages relative to control of P-ERK1/2 protein levels normalized to total ERK1/2 in SVZ cultures. Below the graph, a representative Western blot for 44/42 kDa P-ERK and ERK is shown. N = 5. *P<0.05 using Bonferroni’s multiple comparison test for comparison with the respective controls (TIF) [file pone.0063529.s004.tif]

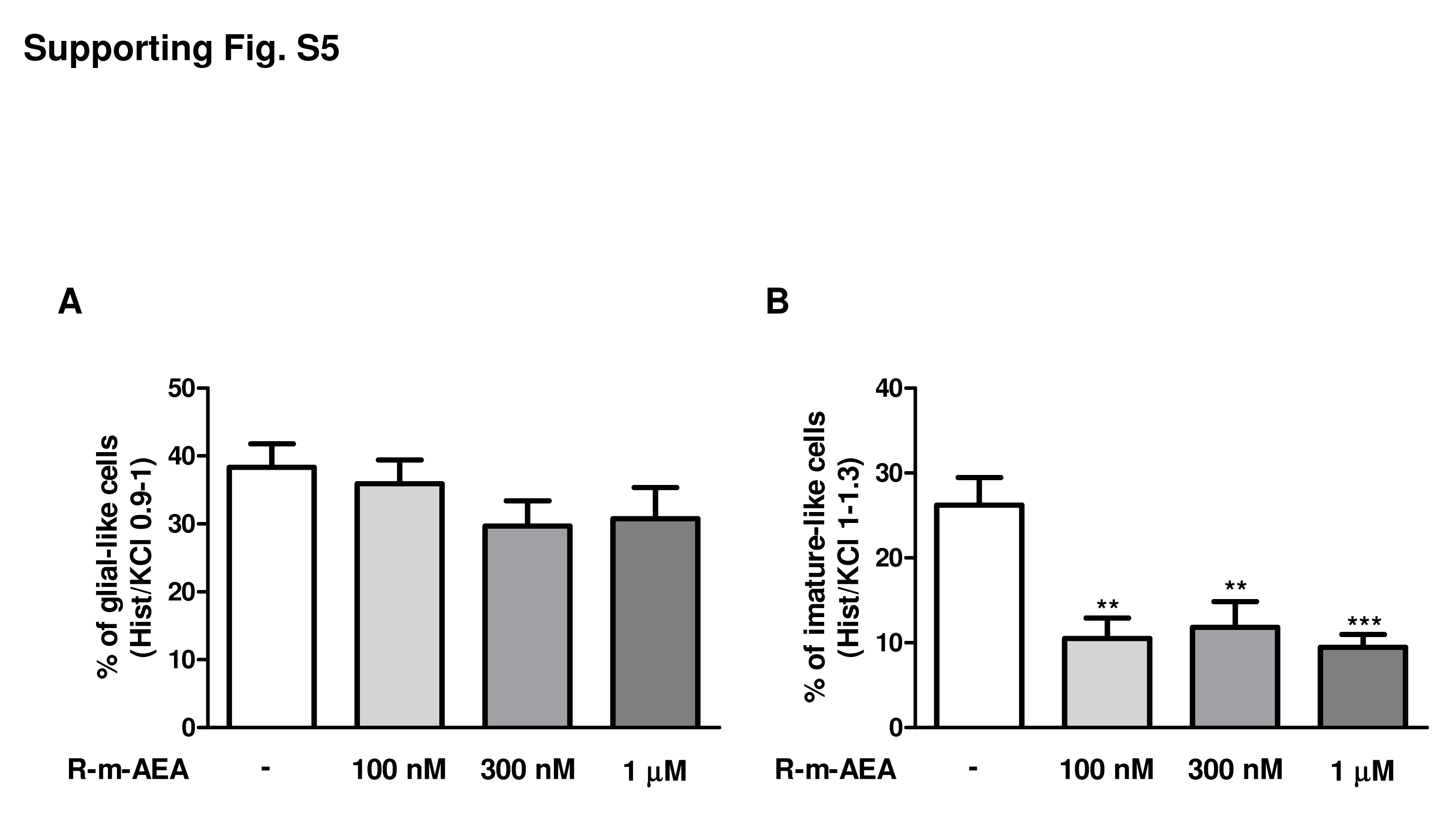

Supplement: Figure S5 — (R)-(+)-Methanandamide does not induce glial differentiation in SVZ cultures through CB1R activation. A: Bar graph depicts the number of glial-like (A) and immature-like (B) responding cells expressed as percentages of total cells analyzed by SCCI. N = 8. **P<0.01 and ***P<0.01 using Dunnett’s multiple comparison test, for comparison with control. (TIF) [file pone.0063529.s005.tif]
