# Supplementary material for: Activation of Type 1 Cannabinoid Receptor (CB1R) Promotes Neurogenesis in Murine Subventricular Zone Cell Cultures
Source: PLoS One. 2013 May 21;8(5):e63529. doi: 10.1371/journal.pone.0063529 (PMC3660454; doi:10.1371/journal.pone.0063529)
Supplement: Methods S1 — Detailed description of methods for: a) SVZ cell cultures; b) single-cell calcium imaging (SCCI); c) quantitative chromatin immunoprecipitation (qChIP); d) cDNA synthesis and realtime RT-PCR analysis (Ngn1). (DOC) [file pone.0063529.s006.doc]

**SUPPORTING INFORMATION**

**SVZ cell cultures:**

Briefly, mice were killed by decapitation, brains were removed and placed on Hank’s balanced salt solution (HBSS) medium supplemented with 100 U/mL penicillin and 100 µg/mL streptomycin (all from Invitrogen, Carlsbad, CA). Fragments of SVZ were dissected out of 450 µm thick coronal brain sections, digested in 0.025 % trypsin and 0.265 mM EDTA (Invitrogen), and dissociated by gentle mixing. Mechanical dissociation was performed with a P1000 pipette and the cell suspension was diluted in serum-free medium (SFM) composed of Dulbecco's modified eagle medium [(DMEM)/F12 + GlutaMAXTM-I)] supplemented with 100 U/ml penicillin, 100 µg/ml streptomycin, 1% B27, 10 ng/ml epidermal growth factor (EGF), and 5 ng/ml basic fibroblast growth factor-2 (FGF-2) (all from Invitrogen). Single cells were then plated on uncoated Petri dishes at a density of 3000 cells/cm^2^. The neurospheres were allowed to develop in a 95% air and 5% CO_2_ humidified atmosphere at 37°C (proliferation conditions). Six days-old neurospheres were adhered for 48h onto poly-D-lysine (0.1 mg/ml) coated glass coverslips in SFM devoid of growth factors (differentiation conditions).

**SCCI**

To functionally evaluate the differentiation pattern of SVZ cells, we analyzed the variations of intracellular calcium-free levels ([Ca^2+^]_i_) in single cells following stimulation with 50 mM KCl and 100 μM histamine (Sigma-Aldrich,) stimulation. KCl depolarization causes an increase in [Ca^2+^]_i_ in neurons, whereas stimulation with histamine leads to an increase in [Ca^2+^]_i_ in stem/progenitor cells. SVZ cultures were loaded for 40 min with 5 μM Fura- 2/AM (Invitrogen), 0.1% fatty acid-free BSA, and 0.02% pluronic acid F-127 (Invitrogen) in Krebs solution (132 mM NaCl, 4 mM KCl, 1.4 mM MgCl_2_, 1 mM CaCl_2_, 6 mM glucose, 10 mM HEPES, pH 7.4), in an incubator with 5% CO2 and 95% atmospheric air at 37°C. After a 10 min postloading period at RT in the same medium without Fura-2/AM and pluronic acid, to obtain a complete hydrolysis of the probe, the glass coverslip was mounted on an RC-20 chamber in a PH3 platform (Warner Instruments; Hamden, CT, USA) on the stage of an inverted fluorescence microscope (Axiovert 200, Carl Zeiss, Gottingen, Germany). Cells were continuously perfused with Krebs solution and stimulated at deﬁned periods of time by applying high-potassium Krebs solution (containing 50 mM KCl, isosmotic substitution with NaCl) and 100 μM histamine. Solutions were added to the cells by a fast-pressurized (95% air, 5% CO2 atmosphere) (AutoMate Scientific, Inc., Berkeley, CA, USA). The variations of [Ca^2+^]_i_ were evaluated by quantifying the ratio of the fluorescence emitted at 510 nm following alternate excitation (750 msec) at 340 and 380 nm, using a Lambda DG4 apparatus (Sutter Instrument, Novato, CA, USA) and a 510 nm band-pass filter (Carl Zeiss) before fluorescence acquisition with a 40x objective and a CoolSNAP digital camera (Roper Scientific, Tucson, AZ, USA). Acquired values were processed using the MetaFluor software (Universal Imaging Corp, Marlow, UK). KCl and histamine peaks given by the normalized ratios of ﬂuorescence at 340/380 nm, at the proper time periods, were used to calculate the ratios of the responses.

**Quantitative chromatin immunoprecipitation (qChIP)**

After treatment of SVZ cells with 1 µM R-m-AEA for 6h and 24h, cells were washed with 0.15 M PBS and the chromatin was crosslinked to proteins by incubating the cells with 1% formaldehyde in PBS containing 50 mM MgCl_2_, for 15 min, at 37°C. SVZ cells were then incubated with 125 mM glycine, for 10 min at RT, washed twice with sterile 0.15M PBS and incubated with lysis buffer [1% SDS, 10 mM EDTA, 50 mM Tris pH 8.1, containing a cocktail of proteinase inhibitors (Roche)], for 5 min.

Samples were sonicated repeatedly on ice (using Sonics & Materials, Model Vibra Cell (TM) VC 50, Parameters: power 20, time 10 x 30 sec with 30 sec intervals, on ice), to cleave genomic DNA. After the analysis of the genomic DNA fragments (of about 1 kbp to 500 bp) in a 1% agarose gel, cell suspension was centrifuged at 10,000xg, for 5 min, at 4ºC. The supernatant was diluted ten-fold in ice-cold dilution buffer containing 0.01% SDS, 1% Triton, 1.2 mM EDTA, 16.7 mM Tris pH 8.1, 167 mM NaCl and a cocktail of protease inhibitors (Roche). Then, samples were precleared with 30 μl of 50% blocked protein G-sepharose beads in 0.15M PBS, for 30 min at 4°C, with rotation. Beads were afterwards eliminated by centrifugation at 10,000xg, for 5 min, at 4ºC. 1/10th was kept as the input, the remaining portion, for each sample, was incubated with 10 μg mouse monoclonal antibody against Histone H3 (tri methyl K36) (H3K36me3; Abcam, Cambridge, UK), at 4°C, overnight. Immune complexes were captured by 1 h incubation with 40 μl of 50% blocked protein G sepharose beads, at 4°C. Then, protein G-sepharose beads were rinsed 7 times, for 10 min, in washing buffer (TE containing 150 mM NaCl and 0.5% NP40) and incubated with 50 μg/ml RNaseA/TE, for 30 min, at 37°C. The input-lysate was prepared in the same way. Proteinase K and SDS were added to 1 mg/ml and 0.5 %, respectively, and samples were incubated overnight at 37°C, before being incubated at 65°C for 3 h to reverse crosslinking. After a centrifugation at 1,000xg, for 5 min, beads were discarded and the DNA samples were purified by phenol-chloroform extraction and precipitated with 1 μg glycogen, plus sodium acetate and ethanol. The DNA pellets were suspended in nuclease-free water and DNA was then subjected to qPCR analysis with SybrGreen (iQ SYBR Green Supermix; Bio-Rad Laboratories, Hercules, CA).

For gene expression analysis, 2 µL of sample DNA or 1 µL of the respective input were added to 10 µL SYBR Green Master Mix (Bio-Rad, 2x concentrated), and the final concentration of each primer was 10 µM in 20 µL total volume. The thermocycling reaction was initiated with activation of Taq DNA polymerase by heating at 95ºC during 3 min, followed by 40 cycles of a 10 sec denaturation step at 95ºC, a 30 sec annealing and elongation step at 64ºC. The fluorescence was measured after the extension step by the iQ5 Multicolor Real-Time PCR Detection System (Bio-Rad). After the thermocycling reaction, a melting curve was performed with slow heating, starting at 55ºC and with a rate of 0.5ºC per 10 sec, up to 95ºC, with continuous measurement of fluorescence, allowing detection of possible nonspecific products. The assay included a non-template control (sample was substituted by sterile water) and a standard curve (in 10-fold steps) of DNA for assessing the efficiency of each set of primers. All reactions were run in duplicates. Primers used in real-time analysis were as follows: *GAPDH*: forward primer 5’-CAA GGC TGT GGG CAA GGT-3’ and reverse primer 5’-TCA CCA CCT TCT TGA TGT CAT CA-3’; *Neurogenin1 (Ngn1)* forward primer 5’-ATT ACG GGC ACG CTC CAG-3’, reverse primer 5’-CAG CTC CTG TGA GCA CCA AG- 3’.

The threshold cycle (Ct) was measured in the exponential phase and therefore was not affected by possible limiting components in the reaction. For every run performed, Ct was set at the same fluorescence value. Data analysis was performed with Biorad iQ5 software. Recovery of genomic DNA as a percentage input was calculated as the ratio of copy numbers in the precipitated immune complexes to the input control. The relative expression of each gene was normalized against GAPDH.

**cDNA synthesis and realtime RT-PCR analysis (*Ngn1*)**

Total RNA was isolated from SVZ cells that were treated with 1 µM R-m-AEA for 3 days and 4 days, according to illustra RNAspin Mini RNA Isolation Kit manufacturer’s instructions (GE Healthcare Life Sciences). The full content of a 6-well culture plate was pooled for each experimental condition. Precipitated RNA was resuspended in 40 µl of diethylpyrocarbonate (DEPC) treated water, and samples were stored at -80ºC until further use. For cDNA synthesis, 1 μg of total RNA was mixed with 2.5 *μ*M anchored-oligo-p(dT)18 primers, 1x PCR reaction buffer, 20 U RNAse inhibitor, dNTPs (1 mM each) and 10 U AMV Reverse Transcriptase in a 20 µl final volume (Roche Molecular Biochemicals). The reaction was performed at 55ºC for 30 min and stopped by 85ºC for 5 min step. The samples were then stored at -80ºC until further use.

For gene expression analysis, 2 µL of sample cDNA were added to 10 µL SYBR Green Master Mix (Bio-Rad, 2x), and the final concentration of each primer was 10 µM in 20 µL total volume. The thermocycling reaction was initiated with activation of Taq DNA polymerase by heating at 94ºC during 3 min, followed by 40 cycles of a 15 sec denaturation step at 94ºC, and a 30 sec annealing and elongation step at 64ºC. Validated primer sets for use in SYBR Green-based real-time RT-PCR were obtained from selected QuantiTect Primer Assays (Qiagen, Valencia, CA). The fluorescence was measured after the extension step by the iQ5 Multicolor Real-Time PCR Detection System (Bio-Rad). After the thermocycling reaction, the melting step was performed with slow heating, starting at 55ºC and with a rate of 0.5ºC per 10 sec, up to 95ºC, with continuous measurement of fluorescence, allowing detection of possible nonspecific products. The assay included a non-template control (sample was substituted by sterile water) and a standard curve (in 10-fold steps) of DNA for assessing the efficiency of each set of primers. All reactions were run in duplicates. Ct values were measured in the exponential phase and were set at the same fluorescence value in each run performed. Data analysis was performed with Biorad iQ5 software (Biorad). The relative expression of each gene was normalized against *GAPDH*, using the comparative Ct method as described by Pfaffl’s formula (Pfaffl 2001).

Supporting References

Pfaffl M. W. (2001) A new mathematical model for relative quantification in real-time RT-PCR. Nucleic Acids Res. 29, e45.
